# Medical Gymnastics and Massage
**Text-book of Massage and Remedial Gymnastics*. By L. L. Despard. Second Edition. (London: Henry Frowde, and Hodder and Stoughton. 1914. Pp. 413 + xix. Price 12s. 6d. net.)


**Published:** 1915-02-20

**Authors:** 


### Medical Gymnastics and Massage.*

At first sight this somewhat formidable volume seems out of proportion to the subject which it proposes to compass. But examination soon shows that its first 210 pages are occupied by a description of the elementary anatomy and physiology of the human body, leaving the remaining 200 for a description of the practice of massage and gymnastics, electrical methods, and ionic medication. As a knowledge of anatomy and physiology is always of interest and value, the earlier part of the book may be read with advantage by anyone desiring to obtain such knowledge, and this more especially as the chapters are well written and are very successfully illustrated. But for our own part we should question whether there is any natural or inevitable relation between the operations of massage and the information that the inferior border of the vomer "articulates with the nasal crest of the maxillæ and palatine bones," or with the announcement that "the nerve supply of the pancreas comes from the hepatic and splenic plexuses." By all means let us have good masseurs and masseuses, but protect us from those whose minds are filled with empty phrases and whose tongues distil words without knowledge. The second part of the book describes the methods of massage and remedial exercises, and applies these in great detail to various diseased conditions, ranging from, say, insomnia to hæmorrhoids. The instruction given is very precise and exact, and on the whole it is well considered and wise. The short descriptions of the symptoms of the various diseases, allowing that such descriptions are necessary or advisable, are very effective, but necessarily suffer from the defects of compression; and in some places definitions are quoted from selected medical writers with a parade which the information afforded hardly seems to justify. In so far as its proper subject-matter is concerned we have nothing but praise for the book, and even where, as we think, it sins by excess it is never marked by carelessness or inaccuracy.

* *Text-book of Massage and Remedial Gymnastics.* By L. L. Despard. Second Edition. (London: Henry Frowde, and Hodder and Stoughton. 1914. Pp. 413 + xix. Price 12s. 6d. net.)

